# Molecular cloning and functional characterization of an ATP-binding cassette transporter OtrC from *Streptomyces rimosus*

**DOI:** 10.1186/1472-6750-12-52

**Published:** 2012-08-20

**Authors:** Lan Yu, Xiangyun Yan, Long Wang, Ju Chu, Yingping Zhuang, Siliang Zhang, Meijin Guo

**Affiliations:** 1 State Key Laboratory of Bioreactor Engineering, East China University of Science and Technology, 130 Meilong Road, Shanghai, 200237, P.R. China; 2 School of Life & Science, Huzhou Teachers College, Zhejiang, Huzhou, 313000, P.R. China

**Keywords:** *Streptomyces rimosus*, OtrC, ATP-binding cassette transporter, ATP hydrolysis, Multidrug resistance

## Abstract

**Background:**

The *otrC* gene of *Streptomyces rimosus* was previously annotated as an oxytetracycline (OTC) resistance protein. However, the amino acid sequence analysis of OtrC shows that it is a putative ATP-binding cassette (ABC) transporter with multidrug resistance function. To our knowledge, none of the ABC transporters in *S. rimosus* have yet been characterized. In this study, we aimed to characterize the multidrug exporter function of OtrC and evaluate its relevancy to OTC production.

**Results:**

In order to investigate OtrC’s function, *otrC* is cloned and expressed in *E. coli* The exporter function of OtrC was identified by ATPase activity determination and ethidium bromide efflux assays. Also, the susceptibilities of OtrC-overexpressing cells to several structurally unrelated drugs were compared with those of OtrC-non-expressing cells by minimal inhibitory concentration (MIC) assays, indicating that OtrC functions as a drug exporter with a broad range of drug specificities. The OTC production was enhanced by 1.6-fold in M4018 (*P* = 0.000877) and 1.4-fold in SR16 (*P* = 0.00973) duplication mutants, while it decreased to 80% in disruption mutants (*P* = 0.0182 and 0.0124 in M4018 and SR16, respectively).

**Conclusions:**

The results suggest that OtrC is an ABC transporter with multidrug resistance function, and plays an important role in self-protection by drug efflux mechanisms. This is the first report of such a protein in *S. rimosus*, and *otrC* could be a valuable target for genetic manipulation to improve the production of industrial antibiotics.

## Background

Clinical multidrug resistance (MDR) to bactericidal antibiotics is driving research into the underlying mechanisms of resistance and the discovery of novel drugs. In the past decade, numerous resistance proteins have identified, including the ubiquitous ATP-binding cassette (ABC) family of multidrug transporters [1-6]. These integral membrane proteins mediate the active extrusion of antibiotics and toxic compounds from cells. Recent crystal structure analyses revealed that some ABC-type multidrug proteins, such as MsbA from *E. coli*, *Vibrio cholera,* and *Salmonella typhimurium*[5-7] and BtuCD from *E. coli*[8], also mediate the import of substrates by altering the conformation of the transmembrane helices (TMs) from an ‘outward-facing’ state to an ‘inward-facing’ state [9]. The ABC transporters represent 2% of gene products in bacteria and play a critical role in their adaptation to the environment [10]. Some are involved in resistance to chemotherapy and, therefore, applied research has increasingly focused on the elucidation of the transportation mechanism of these proteins [11]. Furthermore, the self-protection afforded by the antibiotic producers is dependent on its endogenous drug resistance proteins. Therefore, these resistance proteins may also represent targets for antibiotic production improvement strategies [12,13].

*Streptomyces rimosus*, well known as an oxytetracycline (OTC) producer, has been investigated at the genetic level over many years, although only two antibiotic pump genes have been discovered to date: the OTC resistance gene, *otrB*[14] and a putative ABC transporter gene, *otrC*, which has been isolated, sequenced and deposited in GenBank (AY509111.1). The two open read frames (orf) of the *otrC* gene encode a putative ABC transporter ATP-binding subunit and a putative transmembrane subunit. OtrC was considered to be a putative ABC transporter with an OTC resistance function based on its amino acid sequences, although no evidence in support of this has been reported to date. Generally, the location of resistance genes in the chromosome is close to the corresponding antibiotic biosynthesis clusters. In the case of OTC, the OTC cluster in *S. rimosus* is flanked by the resistance genes, *otrA* and *otrB*[15], while *otrC* is distant from the OTC cluster (data not published). We hypothesize that *otrC* plays a role as the MDR transporter in OTC resistance. Here, we present evidence that *otrC* encodes a protein with ATPase and multidrug efflux activities and therefore may represent a manipulation target for strategies to improve of OTC production in *S. rimosus*.

## Results

### Conserved motifs analysis and model prediction of *otrC*

The *otrC* gene contains two open reading frames (ORFs), which encode a putative ATP-binding subunit (OtrC-ORF1) of 38 kDa and a putative transmembrane subunit (OtrC-ORF2) of 30 kDa. A NCBI BLAST search of the amino acid sequence of the OtrC protein identified a number of similar proteins, the majority of which were ABC components of doxorubicin or MDR ABC transporters from various phyla.

**Figure 1:**
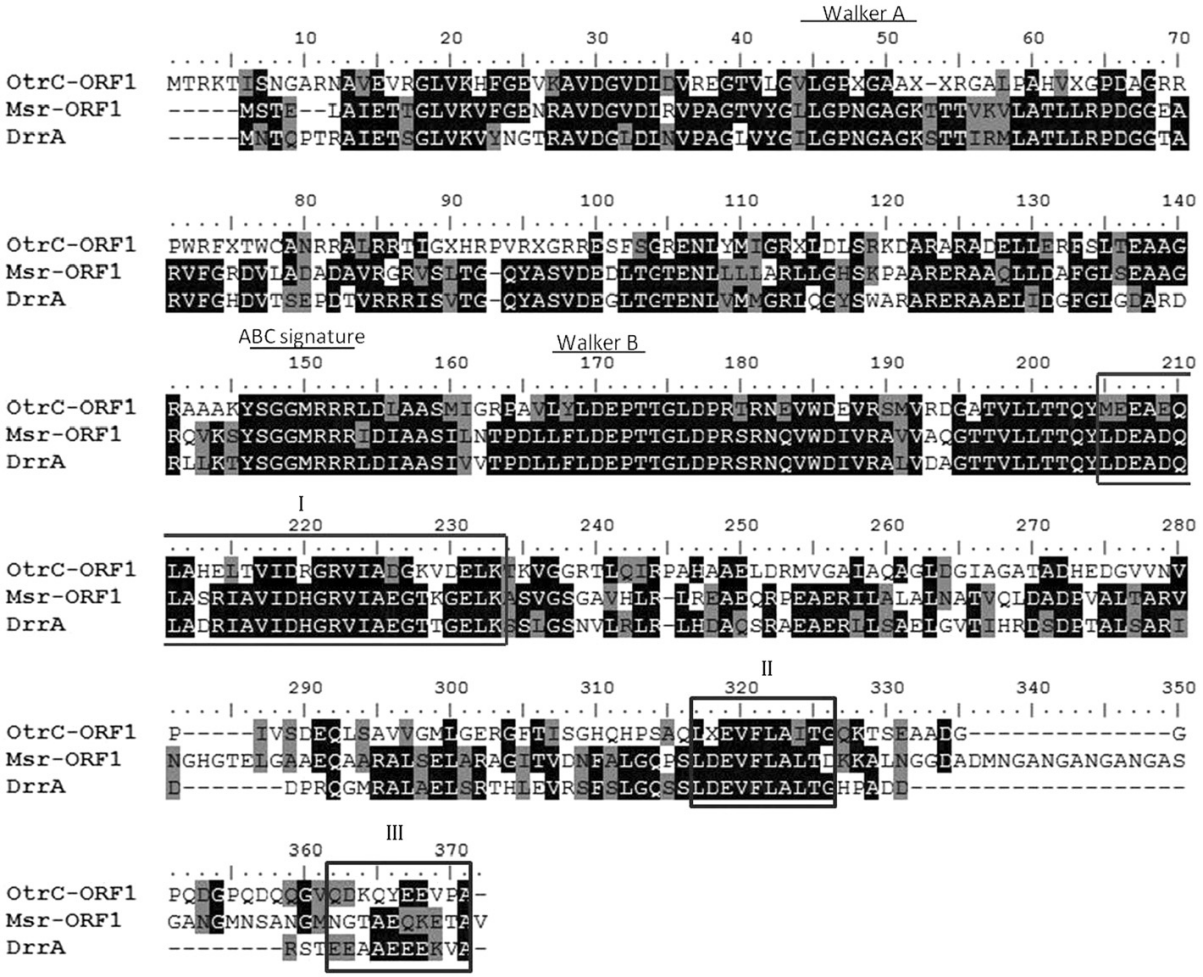
**Alignment of the amino acid sequence of the OtrC-ORF1 region with DrrA and Msr-ORF1.** The dark gray regions show residues that are highly conserved, whereas the residues in light gray are less well conserved.

The ATP-binding subunit usually contains conserved sequence motifs that play important roles in protein function. Therefore, an alignment of OtrC-ORF1 with DrrA [16] and Msr-ORF1 from *Streptomyces*[17] was performed for the identification of conserved motifs (Figure 1). The results showed that OtrC-ORF1 exhibits 41% amino acid identity with the doxorubicin resistance protein DrrA from *S. peucetius* and 43% identity with the multidrug ABC transporter ATP -binding subunit Msr-ORF1 from *S. rochei* F20. The N-terminal of OtrC-ORF1 contains the well-described conserved Walker A and B motifs and the ABC signature sequence. These motifs are intimately involved in ATP hydrolysis [18], which indicates that OtrC belongs to the ABC transporter superfamily. The alignment also showed the conserved LDEADQLA and LDEVFL motifs in the C-terminal region of OtrC-ORF1. These motifs exhibit 58.6% and 80% identity with those of DrrA and Msr-ORF1, respectively. However, the CREEM motif was absent from OtrC-ORF1 and Msr-ORF1. The LDEVFL motif is known to be involved in the doxorubicin-stimulated nucleotide-binding function and also exists in the MDR protein [18]. Therefore, it was speculated that the ATPase activity of OtrC-ORF1 is stimulated by doxorubicin and other drugs.

**Figure 2:**
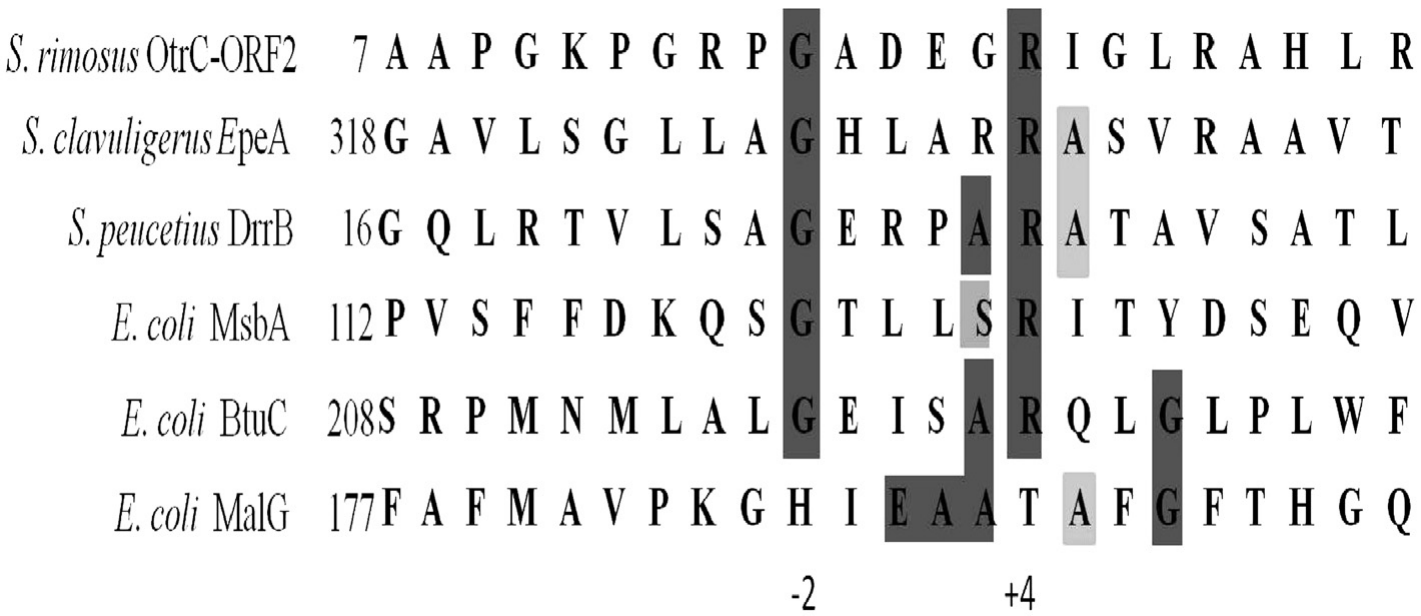
**Alignment of the amino acid sequence of predicted conserved motifs of the transmembrane subunit, OtrC-ORF2, MsbA, DrrB, BtuC and MalG.** The dark gray regions show residues that are highly conserved, whereas the residues in light gray are less well conserved. The first amino acid in the sequence of each protein is shown.

In contrast, the transmembrane subunit has more sequence diversity and varies in the total number of transmembrane helices. OtrC-ORF2 was then aligned with other ABC transporters (Figure 2). The conserved EAA motif (E_1_AA_3_RALG_7_) that is present in MalG was believed to be present only in the importers of the ABC family, while the G_−2_E_−1_…A_3_R/K_4_…G_7_ motif present in BtuC, which has similarity with EAA motif, has been identified in both the import and export functions of ABC transporters [19]. The alignment analysis revealed the present of a G_−2_….R_4_ motif that was present in all ABC transporter proteins with an exporter function, including EpeA, DrrB, MsbA and BtuC. The G_−2_ residue has been identified to correspond with drug sensitivity by site-directed mutagenesis [19]. We therefore assumed that the G_−2_….R_4_ motif was involved in the drug export function of the ABC transporter and OtrC functions as a drug exporter.

The prediction of the secondary structure of OtrC-ORF2 by the PROF method using the PredictProtein program ( http://www.predictprotein.org) indicated the presence of a N-terminal hydrophobic domain with six putative α-helical transmembrane segments and cytoplasmic facing N and C termini. The reported canonical ABC systems display a modular architecture composed of two nucleotide-binding domains/subunits and two transmembrane domains/subunits. Therefore, it can be concluded that the OtrC system is a tetrameric complex composed of two homodimeric constituents, similar to that of many other bacterial ABC transporters [17].

### Functional characterization of OtrC

**Figure 3:**
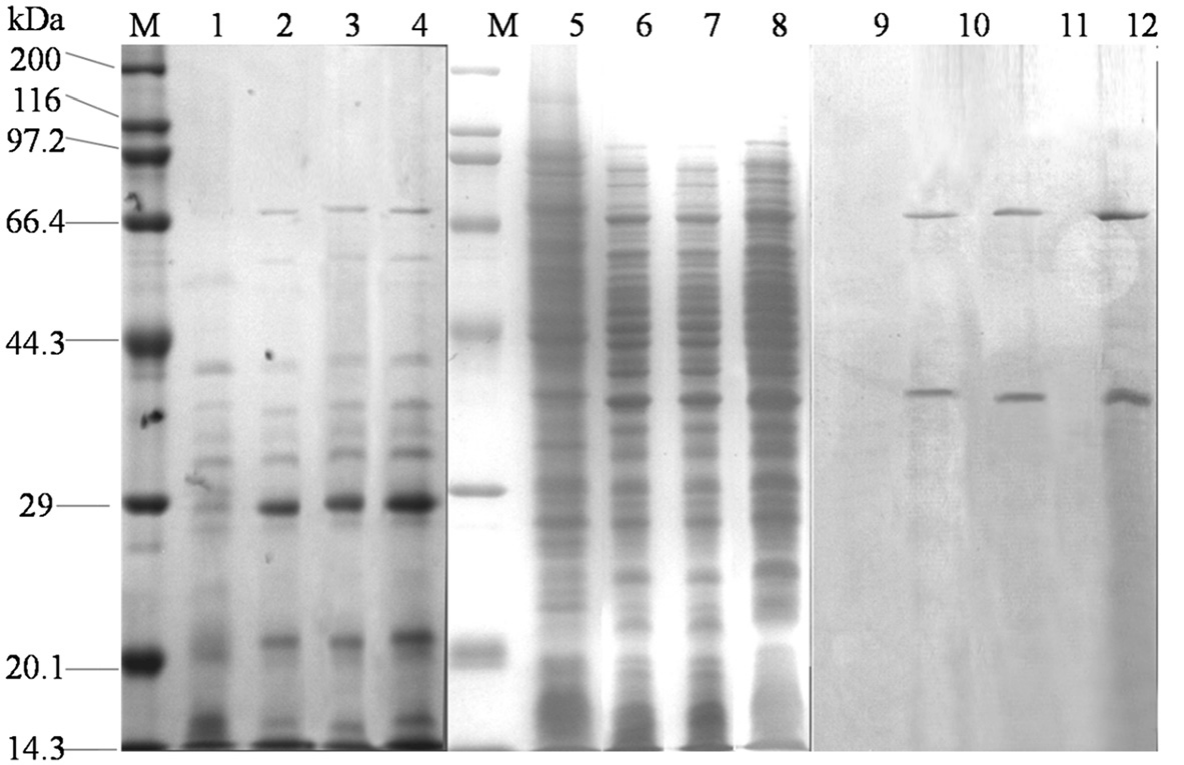
**OtrC heterologous expression in *E. coli.*** Cultures of cells were grown and induced by 1 mM IPTG as described in the Materials and Methods. Cells were resuspended in 500 mM Tris–HCl (pH 7.0) buffer, and cell disruption was performed by ultrasonic waves; the total membrane fractions were harvested by centrifugation at 125,000 rpm at 4°C for 1 h. 12% SDS-PAGE analysis was performed with a high-molecular-weight protein marker (M), 80 μg total membrane fractions of *E. coli*/pET28a (lane 1), 40 μg, 60 μg and 80 μg total membrane fractions of *E. coli*/pETC02 (lane 2,3 and 4), 120 μg total cell protein of *E. coli*/pET28a (lane 5), 60 μg,80 μg and 120 μg total cell protein of *E. coli*/pETC02 (lanes 6,7 and 8). Western blot analysis was performed with equal volumes of total cell protein of *E.coli*/pET28a (lane 9) and *E. coli*/pETC02 (lanes 10, 11 and 12), His-tag mouse and peroxidase-conjugated goat anti-mouse antibodies were used for specific band detection.

*E. coli* BL21 (DE3) cells were transformed with the indicated plasmid pETC02 (Additional file 1: Figure S1), while OtrC expression was induced by IPTG and analyzed by Coomassie blue staining of the SDS-polyacrylamide gels (SDS-PAGE) and Western blotting. A band that corresponded to the 30-kDa OtrC-ORF2 was detected in the cytoplasmic membrane by SDS-PAGE (Figure 3); however, although the OtrC-ORF2 was fused with a His_6_-tag at the C terminal, the specific band of the gene product was absent in the Western blot analysis. This could have been due to the embedding of the His_6_-tag by protein folding. The specific band that corresponded to the 68 kDa-OtrC, 38 kDa-OtrC-ORF1 subunit could be visualized in whole cell protein fraction by SDS-PAGE and Western blot analysis (Figure 3). These specific bands of OtrC and its subunits were absent from *E. coli* that carried the empty pET28a plasmid, which suggested the successful heterologous expression of OtrC in *E. coli*.

**Figure 4:**
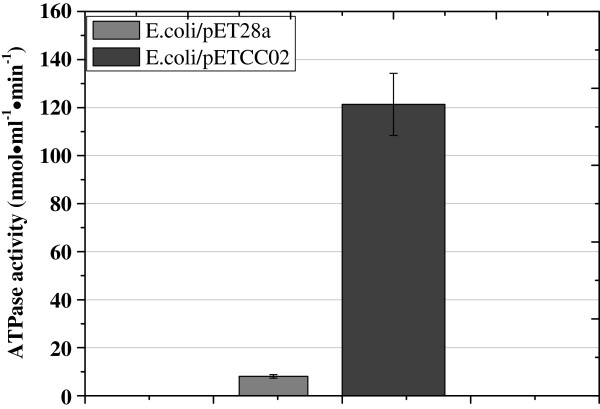
**ATPase assay of OtrC-overexpressing and OtrC-nonexpressing cells. ***OtrC* from *S. rimosus* was introduced into the *E.coli* BL21(DE3) strain using a pETC02 plasmid, and the *E.coli* BL21(DE3) carrying the empty pET28a was used as the control. The cells were induced by 1 mM IPTG at 30°C for 10 h, and then collected by centrifugation. The cell wall was digested by lysozymes and the membrane vesicles were harvested by centrifugation. The ATPase activity of *E.coli*/pETC02 (dark gray) was determined compared with to the *E. coli*/pET28a control (light gray). Vertical error bars corresponded to the standard error of the mean of three replicated samples.

The malachite green assay was employed to determine the ATPase activity of OtrC. The results showed that the ATPase activity of *E. coli/*pETC02 (121.3 nM·ml^-1^·min^-1^) was significantly higher than that of *E. coli/*pET28a (8.1 nM·ml^-1^·min^-1^) (Additional file 4: Figure S2 and Figure 4), which indicates as expected that OtrC has ATP hydrolysis activity.

**Figure 5:**
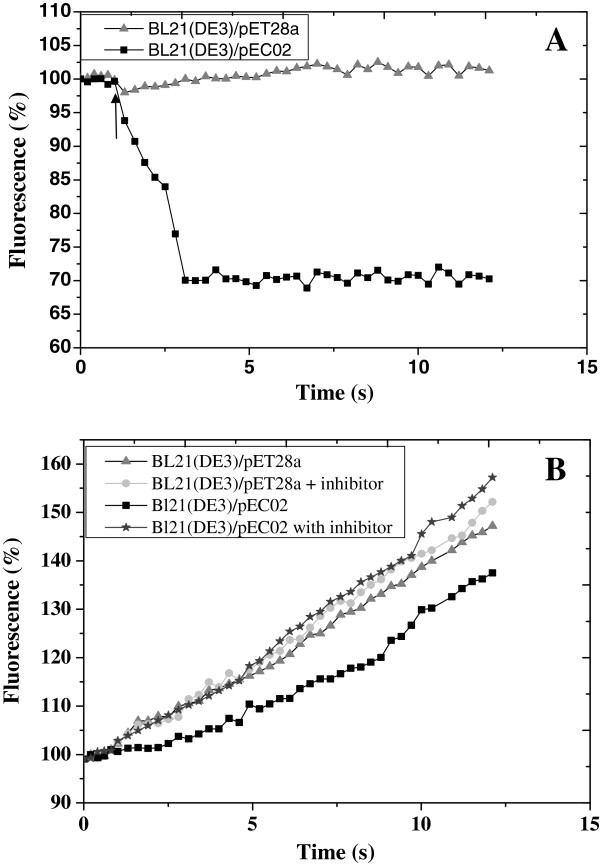
**Ethidium bromide (EB) transport in OtrC-overexpressing cells and nonexpressing cells. A**: The OtrC-expressing cells (black squares) and the control cells (gray triangles) were cultured and induced with 1 mM IPTG, and then resuspended in KPi buffer (pH 7.0) containing 5 mM MgSO_4_. The de-energized cells (OD_600_ = 0.5) were pre-equilibrated with 20 μM EB at 30°C, and 25 mM glucose was added (at the arrow) to initiate EB efflux. **B**: The effect of orthovanadate on the accumulation of EB in OtrC-expressing and nonexpressing cells. *E.coli*/pETC02 cells were cultured, induced and collected; the washed cells (OD_600_ = 0.5) were incubated for 10 min at 30°C in the presence (dark gray stars) or absence (black squares) of 0.5 mM orthovanadate, followed by the addition of 20 μM ethidium bromide (EB) along with 2 mM Mg-ATP (arrow). The *E. coli*/pET28a cells were used as the control, treated with (grey triangles) or without (light gray circles)orthovanadate as described above. The development of fluorescence of the DNA-ethidium complex in the cell suspension was monitored at 30°C every 20 s. The fluorescence intensity before the addition of glucose or ATP was normalized to 100%.

Fluorimetric EB, a cationic substrate of ABC transporters, was employed for the efflux assays to test if OtrC can mediate the transport coupled to ATP hydrolysis. The cells of *E. coli* transformants were suspended in 20 μM EB solution, and the EB efflux activity was triggered by glucose. As shown in Figure 5A, the energization of cells resulted in an increased rate of EB extrusion for OtrC-expressing cells compared to the control cells, which indicates that OtrC displays an ATP-dependent extrusion activity. The efflux activity of OtrC was further confirmed by determining the effect of an ABC transporter inhibitor orthovanadate on the accumulation of EB in OtrC-expressing and OtrC-nonexpressing cells. As the glycolysis pathway also can be inhibited by orthovanadate, cells were energized with ATP instead of glucose. As shown in Figure 5B, orthovanadate increased the level of EB uptake in OtrC-expressing cells, while no increase was observed in OtrC-nonexpressing cells. These observations indicate that orthovanadate inhibited the OtrC-mediated efflux of EB.

### Effect of OtrC expression on drug susceptibility

In order to identify the MDR function of OtrC, the OtrC-overexpressing and non-expressing mutants of the *S. rimosus* M4018 strain were constructed and identified as described in the Materials and Methods (Additional file 1: Figures S3 and S4).

Several drugs with clinical importance were employed for drug susceptibility assays of OtrC-overexpressing and OtrC-nonexpressing strains of *E. coli* as well as *S. rimosus*. As shown in Tables 1 and 2, the MIC for ampicillin increased by 1.3-fold, and in the order of 3.3 fold for doxorubicin, 2-fold for EB and vancomycin in *E. coli/*pETC02 compared with *E. coli/*pET28a. The MICs of *E. coli*/pETC02 to OTC and ofloxacin were enhanced at 5 μg/ml and 2 μg/ml, respectively; in contrast, the growth of *E. coli/*pET28a was completely inhibited by these drugs. However, the overexpression of OtrC had no effect on the susceptibility of *E. coli* to rifampicin, erythromycin and streptomycin (Table 1).

**Table 1:** **Susceptibilities of *E. coli *mutants to several drugs**

**Antibiotics (μg/ml)**	**BL21(DE3)/pET28a**	**BL21(DE3)/pETC02**
Ampicillin	40	50
Rifampicin	40	40
Oxytetracycline	-	5
Doxorubicin	150	500
Ethidium bromide	200	400
Vancomycin	2	4
Ofloxacin	-	2
Erythromycin	10	10
Streptomycin	15	15

‘-’, cell growth was completely inhibited.

**Table 2:** **Susceptibilities of *S. rimosus *mutants to several drugs**

**Antibiotics (μg/ml)**	**M4018/pSET152**	**M4018/pSEC**	**M4018/pKCΔ*otrC***
Ampicillin	500	1500	100
Rifampicin	200	200	200
Oxytetracycline	1500	2500	1000
Doxorubicin	500	1000	100
Ethidium bromide	4	8	4
Vancomycin	1	500	-
Ofloxacin	20	400	4
Erythromycin	2	2	2
Streptomycin	+	+	+

‘+’, cell growth could not be inhibited by the antibiotic; ‘-’, cell growth was completely inhibited.

A clear difference in drug sensitivity to these drugs was also found in *S. rimosus* mutants M4018/pSEC and M4018/pKCΔotrC with respect to the control strain M4018/pSET152. The overexpression of OtrC in M4018/pSEC resulted in significantly elevated MICs to ampicillin (3-fold), OTC (1.7-fold), doxorubicin (2-fold), EB (2-fold) and ofloxacin (20-fold). Furthermore, the OtrC overexpressing mutant survived with 500 μg/ml vancomycin, while the growth of M4018/pSET152 was completely inhibited by vancomycin (Table 2). The MICs for ampicillin and doxorubicin in the OtrC-disrupting mutant M4018/pKCΔotrC were decreased by 20%, compared to a decrease of 67% for OTC; however, the disruption of OtrC had no effect on EB and vancomycin sensitivity (Table 2). Neither the OtrC-overexpressing mutant nor the OtrC-disrupting mutant showed any alternation in the sensitivity to rifampicin, erythromycin and streptomycin (Table 1), which was in agreement with the results of drug susceptibility assays in OtrC-overexpressing *E. c*o*li* cells.

The *otrC* overexpressing cells showed a significantly enhanced MIC to ampicillin, OTC, doxorubicin, EB, ofloxacin and vancomycin, which indicated that OtrC functions as a drug exporter with a broad range of drug specificities.

### OtrC expression affects OTC production in *S. rimosus*

**Figure 6:**
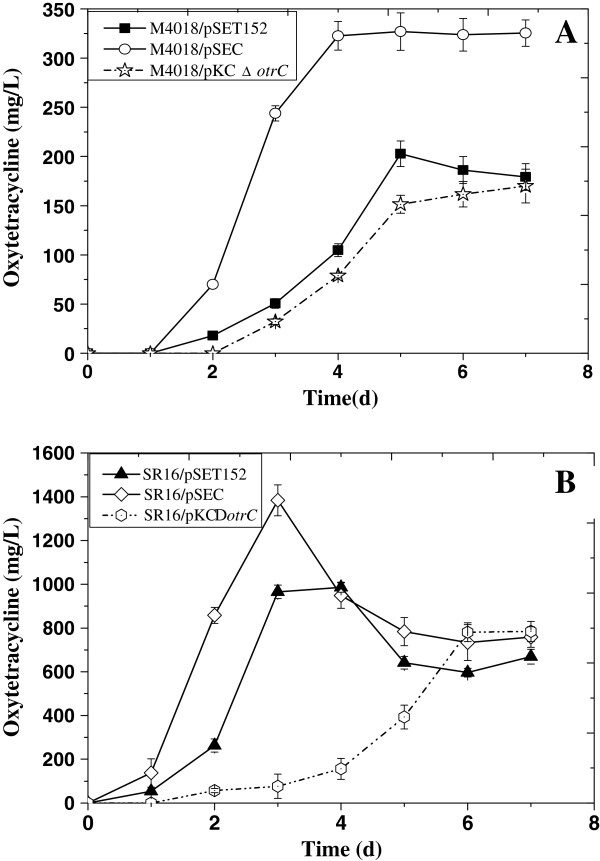
**Specific OTC production of *S. rimosus *strains. A**: An extra copy of *otrC* was introduced into the *S. rimosus* M4018 chromosome using the recombinant integrative vector pSEC (white circles). The *otrC* disruption mutant of M4018 was constructed using the temperature-sensitive recombinant plasmid pKCΔ*otrC* (white stars). M4018/pSET152 (black squares) served as the control. **B**: An extra copy of *otrC* was introduced into the chromosome of *S. rimosus* SR16 using the integrative vector pSEC (white diamonds). The *otrC* disruption mutant of SR16 was constructed using the temperature-sensitive plasmid pKCΔ*otrC* (white hexagons). SR16 that carried the empty pSET152 plasmid was used as the control (black triangles). *S. r*i*mosus* strains were grown in MS plates, spores were inoculated into GYCS medium to the final concentration of 1 × 10^6^ spores per ml, after which they were cultured at 28°C on a rotary shaker (260 rpm) for 72 h; 1% seed culture was transferred into SC medium cultured at 30°C on a rotary shaker (260 rpm) for 7 d. OTC was measured by HPLC. Vertical error bars correspond to the standard error of the mean of three replicated cultures.

In order to explore the applications of OtrC for antibiotic production, the identified recombinant plasmids, pSEC and pKCΔotrC (Additional file 1: Figures S2 and S3), were introduced into an industrial OTC overproducer, SR16, after identification as described in the Materials and Methods. Mutant strains were used for OTC production measurements in addition to the mutant derived from strain type M4018. The results showed that the mutant M4018/pSEC yielded an OTC production peak of 327.0 μg/ml at 96 h, which was markedly higher than that of M4018/pSET152 (202.8 μg/ml at 120 h), while the *otrC* disruption mutant M4018/pKCΔotrC yielded a decreased OTC production peak of 170.0 μg/ml at 168 h (Figure 6A). The OTC production peak in M4018/pSEC at an earlier time than the controls suggested that the OTC efflux function of OtrC plays an important role in self-protective mechanisms and OTC production.

The OTC production profile in SR16 mutants showed that the OTC production of the OtrC knock-in mutant SR16/pSEC reached a peak of 1,384.1 mg/L at 72 h, which was 1.4-fold greater than that of SR16/pSET152 at 96 h. In contrast, the production of OTC was markedly reduced in the disrupted mutant SR16/pKCΔotrC. It yielded an OTC production rate of 784.2 mg/L at 168 h, which was approximately 80% of the peak production of SR16/pSET152 (Figure 6B). These results were in agreement with the above-mentioned conclusion that OtrC expression efficiently affects OTC production in *S. rimosus*.

**Figure 7:**
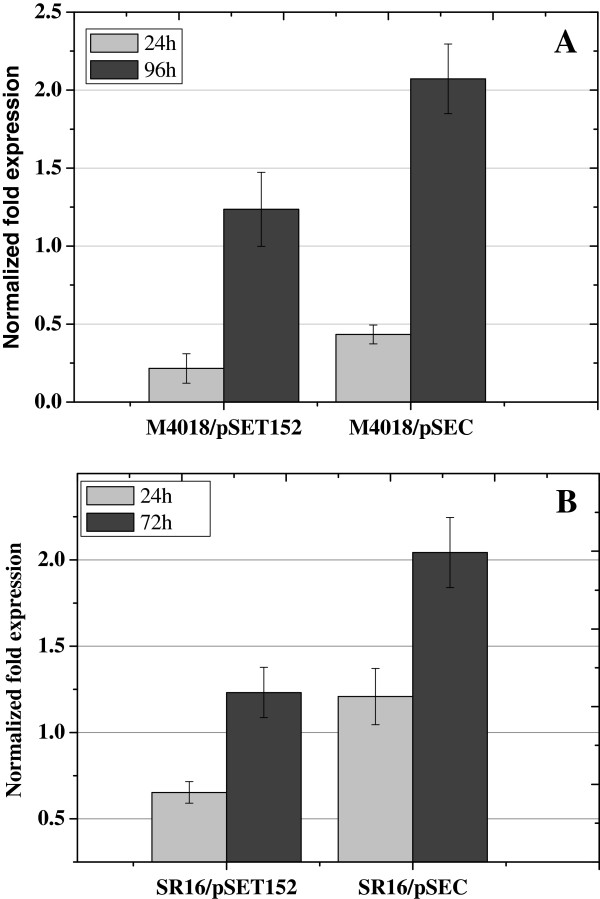
**qRT-PCR analysis of the transcriptional level of *otrC *in *S. rimosus *mutants. A**: The transcriptional level of *otrC* in M4018/pSEC at 24 h (light gray) and 96 h (dark gray) during OTC fermentation, where M4018/pSET152 was used as the control. **B**: The transcriptional level of *otrC* in SR16/pSEC at 24 h (light gray) and 72 h (dark gray) during OTC fermentation, SR16/pSET152 was used as the control. *S. rimosus* cells were collected at different developmental stages during culture in fermentation medium. RNA extraction and quality control is described in the Materials and Methods. Three housekeeping genes, 16S rRNA, *hrdB* and *zwf1*, were used as the reference genes for qRT-PCR analysis.

The transcription levels of *otrC* in *otrC*-expressing *S. rimosus* strains were measured by qRT-PCR. The transcription level of *otrC* was significantly enhanced in *S. rimosus* M4018/pSEC compared with the control strain M4018/pSET152 (Figure 7A), which explains the significant improvement of OTC production in M4018/pSEC. For the industrial overproducer SR16, the *otrC* duplication mutant SR16/pSEC showed a marked enhancement in the transcription of *otrC* compared with the negative control, SR16/pSET152 (Figure 7B). These results suggest that *otrC* overexpression was responsible for OTC overproduction in M4018/pSEC and SR16/pSEC.

## Discussion

The bacterial ATP-binding cassette (ABC) family of multidrug transporters contains very important resistance proteins that have evolved over a long period. In the present study, we characterized the function of OtrC, a putative ABC transporter, from *S. rimosus* and report that OtrC has major effects on the overproduction of OTC in *S. rimosus.*

### The multidrug exporter function of OtrC from *S. rimosus*

**Figure 8:**
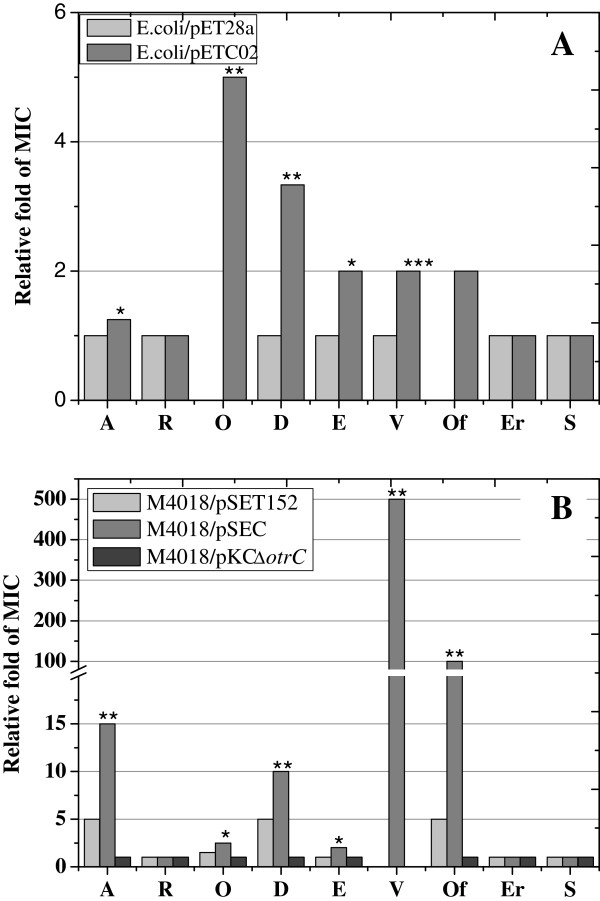
**The relative fold change of MICs of OtrC-expressing cells with different drugs compared to OtrC-nonexpressing cells. A**: The relative change in the MIC of OtrC-expressing *E. coli*/pETC02 cells (gray) to ampicillin (A), rifampicin (R), oxytetracycline (O), doxorubicin (D), ethidium bromide (E), vancomycin (V), ofloxacin (Of), erythromycin (Er) and streptomycin (S), using the OtrC-nonexpressing *E.coli*/pET28a cells (light gray) as the controls. The relative change in MIC to the drug that could completely inhibit the cell growth of *E.coli*/pET28a was set as “0”, while the MIC of drug which could not inhibit the cell growth was set as “1”. Cells were grown in LB liquid medium supplemented with kanamycin, IPTG and different concentrations of test drugs at 37°C, and spun at 170 rpm for 12 h. Cells were then resuspended in fresh LB and spread immediately on the LB plates with kanamycin, IPTG and different concentration of the test drugs. The MICs to drugs were determined after the plates were cultured at 37°C for 12 h. **B**: The relative fold of MICs of OtrC-expressing *S. rimosus* mutants, M4018/pSET152 (light gray) and M4018/pSEC (gray), to the above-mentioned drugs, using the OtrC-nonexpressing mutant M4018/pKCΔ*otrC* (dark gray) as the control. The relative change in the MIC to drugs that could completely inhibit the cell growth of M4018/pKCΔ*otrC* was set as “0”, while the MIC of drug which could not inhibit the cell growth was set as “1”. Cells were grown in TSB liquid medium supplemented with different concentrations of drugs at 28°C, and the centrifuged at 220 rpm for 30 h, resuspended in fresh TSB and spread immediately on TSB plates with different concentrations of test drugs. The MICs of drugs were determined after the plates were cultured at 28°C for 3 d. ** indicate significantly higher MIC values than the control cells; * indicate higher MIC values than the control cells.

The results of the amino acid sequence alignment analysis showed that OtrC contains a N-terminal ATPase activity-related conserved motif and a drug resistance activity-related motif at the C-terminal (Figure 1). As the highly conserved LDEVFL motif of DrrA at the C-terminal was shown to be involved in doxorubicin efflux, this indicated that OtrC might also be associated with antibiotic resistance. The present results (Figure 8) show that drug susceptibility levels to tetracycline, EB and floxacin were enhanced in strains of *E. coli* and *S. rimosus* that overexpressed OtrC compared to corresponding OtrC-nonexpressing strains, which were very similar findings to those published previously regarding DrrA and Msr-ORF1 [4,17]. Additionally, we hypothesized that the low level of conservation of the amino acid sequence in the LDEADQLA motif of OtrC-ORF1 (Figure 1) may be responsible for the consistently low sensitivity to erythromycin of OtrC-expressing cells.

OtrC-ORF2 contains the G_−2_…R_4_ conserved motif, but does not possess the EAA conserved motif (Figure 2). The former might be involved in drug export function, and the latter was identified to be involved in import function [19]. To verify the function of OtrC as a drug transporter in cells, the *E. coli* cells that overexpressed OtrC were given glucose, which resulted in the extrusion of EB rather than its uptake as was expected (Figure 5). Furthermore, when the ABC transporter inhibitor orthovanadate was added, EB extrusion was not observed in the OtrC-expressing cells supplemented with ATP. This indicated that OtrC is responsible for the EB export activity in OtrC-expressing *E. coli* transformant. Hence, the drug resistance mechanism of OtrC could be based on ATP hydrolysis coupled with its transport ability.

The OtrC-expressing *E. coli* transformant showed significantly enhanced susceptibilities to ampicillin, doxorubicin, OTC, EB, vancomycin and ofloxacin (Figure 8A). However, the MICs of *S. rimosus* mutants to ampicillin, OTC, doxorubicin and ofloxacin were markedly amplified compared with controls (Figure 8B), which suggested that OtrC could play an important role in the self-protection mechanisms of *S. rimosus*. Interestingly, the MIC of M4018/pSEC to vancomycin was 500 μg/ml, and the growth of M4018/pKCΔotrC and M4018/pSET152 was completely inhibited under these conditions. This implies that OtrC acts as an efficient vancomycin efflux pump. Additionally, the significantly enhanced vancomycin resistance activity in *S. rimosus* M4018/pSEC might be attributed to the strong constitutive ermE* promoter, which results in the overexpression of OtrC during the early growth phase.

### Effect of OtrC expression on OTC production in *S. rimosus*

The M4018 strain showed a stable production of OTC. In contrast, the SR16 mutants showed an obvious drop of OTC production after 3 days, which was probably due to the instability of SR16 (Figure 7). A red pigment was observed during SR16 fermentation (date not published), which, to our knowledge, has not been previously described in *S. rimosus*. We speculate that another competitive secondary metabolic pathway was activated in SR16 during the traditional mutation.

The real-time qRT-PCR analysis showed that OtrC overexpression could lead to a significant OTC production improvement by secreting OTC out of cells in both the M4018 *S. rimosus* strain and an industrial overproducer, SR16. Hence, it is reasonable to suggest that *otrC* could be a valuable target for genetic manipulation, such as introducing extra copies of the ABC transporter [12,13], overexpressing the pathway genes with the use of transcription regulators [20-22] and the replacement of heterologous promoters [23,24], in order to yield the overproduction of OTC.

## Conclusion

OtrC is a putative ABC-type transporter in *S. rimosus*, although it has been considered to be an OTC resistance protein. However, the drug resistance function and mechanism of OtrC have not been studied previously. In this study, the alignment of the amino acid sequence of OtrC suggested that it could belong to an ABC superfamily and could act as an ATP-binding drug exporter, which was further identified by ATPase activity determination and EB efflux assays in this study. The results of the drug susceptibility tests showed that OtrC confers a functional resistance toward a broad range of structurally unrelated drugs. Hence, OtrC is a multidrug resistance protein based on an ATP hydrolysis-dependent active efflux mechanism. It is the first reported multidrug resistance ABC transporter in *S. rimosus*.

The further investigation of mutations of conserved amino acid residues that inhibit the OtrC pump could be valuable for controlling drug resistance in clinically important pathogens. Improvements in OTC production were successfully made by introducing an extra copy of *otrC* into the high-performance SR16 strain, while the disruption mutant displayed a decreased OTC yield. This highlights the potential value of OtrC in OTC industrial production. Future studies on the OTC efflux activity of OtrC and the effects of OtrC overexpression in *S. rimosus* will contribute to improvements in OTC production.

## Methods

### Bacterial strains, plasmids, antibiotics and antibodies

The bacterial strains used in this study were *E. coli* DH5α, ET12567 (pUZ8002) [25], BL21 (DE3), *S. rimosus* M4018 (a typical strain, gift from Prof. Iain S Hunter, University of Strathclyde, UK), and SR16 (an industrial overproducer obtained by traditional strain improvement, Shanxi Tongxing Antibiotic Company, China).

The plasmids used in this study included pMD19-T, pSET152, pKC1139, pET28a and pSEC (*otrC* in pSET152), pKCΔ*otrC* (for otrC disruption, reconstructed by pKC1139), pETC02 (*otrC* in pET28a). *E. coli* strains were screened using a LB plate with 100 μg·ml^-1^ ampicillin, 50 μg·ml^-1^ apramycin, 25 μg·ml^-1^ kanamycin or 34 μg·ml^-1^ chloramphenicol when appropriate. *S. rimosus* mutants were screened by TSB plates with 500 μg·ml^-1^apramycin or 500 μg·ml^-1^ kanamycin.

Different concentration of ampicillin, oxytetracycline, doxorubicin, ethidium bromide, vancomycin, ofloxacin, rifampicin, erythromycin and streptomycin were used for the MDR assay with OtrC. His-tag mouse and peroxidase-conjugated goat anti-mouse IgG (SAB, USA) antibodies were employed for Western blotting analyses.

### Media and growth conditions

*Escherichia coli* strains were cultured in Luria-Bertani (LB) medium (1.0% tryptone, 1.0% NaCl, 0.5% yeast extract) with appropriate antibiotics when necessary. *S. rimosus* strains were grown in tryptone soya broth (TSB) medium (3% tryptone soya broth, Oxoid) for genomic DNA extraction and mycelium growth, adding appropriate antibiotics, where needed. The mannitol soya flour (MS) medium (2% mannitol, 2% soya flour and 2% agar) was used to grow spores [26]. The seed cultures were obtained by inoculating spores into glucose yeast casein hydrolysate and sucrose (GYCS) medium (1% glucose, 0.05% yeast extract, 1.5% casein hydrolysate, 0.28% sucrose, 0.01% CaCO_3_). Soluble starch and corn steep liquor (SC) medium [27] (2% soluble starch, 1% corn steep liquor, 0.6 (NH_4_)_2_SO_4_, 0.8% CaCO_3_, 0.5% NaCl, 0.2% soy bean oil, pH 6.8 to 7.2).

### OtrC expression in *E. coli*

For OtrC heterologous expression in *E. coli*, the genomic DNA of M4018 was used as the template. The following primers, orf1F (5’CGCCATATGATGAC GCGAAAGACGATATCCA3’) and orf1R (5’CGCGGATCCTCATGCCGGAACCTCCTCG3’), were used to amplify the *otrC* open read frame 1 (*orf1*), which was introduced into the *Nde*I and *Bam*HI sites (underlined); primers orf2F (5’CGCGGATCC**GAAGGAGA**TATACCATGAGTGCCGCGACGGT3’) and orf2R (5’ATTTGCGGCCGCGGTCTTCTTGCGGAACTTGGC 3’) were used to amplify *otrCorf2* which was introduced into the *BamH*I and *Not*I sites (underlined), and the optimized RBS sites of the T7 promoter (bold) was introduced towards the 5’ end. The amplified fragments (*orf1*, 1,074 bp and *orf2*, 870 bp) were subcloned into T-vector for sequencing, and then digested by restriction enzymes and cloned into the pET28a vector under the T7 promoter to construct the recombinant plasmid, pET28a-*otrCorf1-otrCorf2* (pETC02).

After pETC02 was introduced into the *E. coli* BL21 (DE3) strain, for OtrC expression, the *E. coli* transformant (*E.coli*/pETC02) was cultured in LB medium with 50 μg/ml kanamycin. The growth of the cultures was monitored by recording the optical density at 600 nm (OD_600_); when it reached at 0.6, cells were induced by isopropyl beta-D-thiogalactopyranoside (IPTG) at a final concentration of 1 mM at 30°C, at 170 rpm for 10 h. The *E. coli/*pET28 transformant was cultured and induced under the same conditions and used as a negative control.

One-milliliter cell suspensions were centrifuged at 12,000 rpm for 10 min at 4°C, and washed with deionized water, then resuspended in 100 μl Tris–HCl (500 mM pH 7.0) buffer, and the cell disruption was performed by ultrasonic waves. The cell disruption suspension was centrifuged at 12,000 rpm for 15 min at 4°C to remove the cell debris, and the total membrane fractions were then harvested by centrifugation at 125,000 rpm, at 4°C for 1 h. The total protein sample and the membrane fractions were then analyzed by 12% SDS-polyacrylamide gel electrophoresis (SDS-PAGE) and Western blotting following standard procedures [26].

### ATPase assay of OtrC

For the ATPase assay, *E. coli* transformants were cultured in LB with 50 μg/ml kanamycin and induced with IPTG, as described above. A cell suspension sample of 1.5 ml with OD_600_ = 0.6 was obtained, and cells were collected by centrifugation at 10,000 rpm for 1 min. Cells were then washed with deionized water twice. Cell walls were digested by incubation for 1 h at 30°C with 10 mg/ml lysozyme (BBI, UK), after which DNase (100 μg/ml, TaKaRa, Japan) and 10 mM MgSO_4_ were then added. After a 10-min incubation, the suspension was centrifuged at 16,000 rpm for 15 min at 4°C to remove the cellular debris, and the membrane vesicles were then harvested by centrifugation at 125,000 rpm at 4°C for 1 h, and resuspended in 1.5 ml buffer A (50 mM Hepes pH 8.0, 250 mM NaCl, 10% glycerol [v/v]), and used as the protein sample in immediate assays for ATPase activity [1].

The malachite green assay was used to determine the specific ATPase activity of OtrC by measuring the release of inorganic phosphate. Briefly, 2 mM ATP was added into a 200 μl protein sample, 25-μl aliquots were transferred into 175 μl of 10 mM sulfuric acid to stop the reaction for 20 min. Subsequently, 50 μl of fresh malachite green/molybdate solution was added, and the absorbance at 650 nm was measured after incubation for 10–15 min [1,28].

The malachite green/molybdate solution was freshly prepared and it contained malachite green solutions 1 (0.122% [w/v] malachite green, 20% [v/v] sulfuric acid), solutions 2 (7.5% [w/v] ammonium molybdate) and solutions 3 (11% [v/v] Tween 20) at a ratio of 50:12.5:1 [1].

### Efflux assay of OtrC

The *E. coli*/pETC02 was cultured in LB with 50 μg/ml kanamycin and induced with IPTG. Subsequently, the cells were collected by centrifugation at 10,000 rpm at 4°C for 10 min, before being washed with 1 volume 50 mM KPi (pH 7.0) containing 5 mM MgSO_4_. The washed cell suspensions (OD_600_ = 0.5) were incubated for 10 min at 30°C in the presence of 20 μM ethidium bromide (EB). The EB efflux was initiated by the addition of 25 mM glucose, and the cell suspension was used to monitor the fluorescence of DNA-EB complex by a fluorimeter (Cary Eclipse, Australia) at 30°C using excitation and emission wavelengths of 500 and 580 nm, respectively, and slit widths of 5 and 10 nm, respectively [29].

To study the effect of ABC inhibitor *Ortho*-vanadate on the accumulation of EB in OtrC expressing and non-expressing *E. coli* cells [29], cells were cultured, induced, collected and washed as described above. Washed cells (OD_600_ = 0.5) were incubated for 10 min at 30°C in the presence or absence of 0.5 mM orthovanadate, followed by the addition of 20 μM ethidium bromide (EB) along with 2 mM Mg-ATP, and the fluorescence of EB was measured at 30°C as described above.

### OtrC knock-in and disruption in *S. rimosus*

The ermE*p promoter region was amplified by using E*pf (5’CCGGAATTGTACCAGCCCGACCCGAG3’) and E*pr (5’ CGCGGATCCGTGGAGTGGTTCTGTATCCTACCAA 3’) as the primers to amplify a 306 bp fragment encompassing the *Eco*RI and *Bam*HI cloning sites. The entire *otrC* gene (1,895 bp) encompassing the *Bam*HI and *Xba*I sites were amplified using genomic DNA of M4018 as templates and otrCf (5’ CGCGGATCC**CCTCTTACGAGNAAGTC**ATGAAGTTCCGCCGAATGNA 3’,) and otrCr (5’ TGCTCTAGATCAGGTCTTCTTGCGG AACTT3’) primers, where the natural RBS sequence of *otrC* was introduced into primer otrCf (bold). The fragments were cloned into a pMD19-T vector for sequencing, and the fragments were digested by the restriction enzymes after identification and inserted into a pSET152 vector to obtain the recombinant plasmid, pSET152-ermE*p-otrC (pSEC). This was identified by enzyme digesting and sequencing. Plasmid pSEC was introduced into *E. coli* ET12567 (pUZ8002) for demethylation and then introduced into the chromosomes of M4018 and SR16 by electroporation following standard procedures to generate the M4018/pSEC and SR16/pSEC mutant strains. The empty plasmid, pSET152, was introduced into M4018 and SR16 to construct M4018/pSET152 and SR16/pSET152, respectively, which were used as controls.

For the disruption of *otrC*, the CZ1 and CZ2 fragments were amplified by PCR using two sets of primer pairs: CZ_1_f (5’CCGGAATTCTGCCTGCCCGCCGTC3’) and CZ_1_r (5’ CCGGATATCTCCTCGTGGTCGGCGGT 3’), CZ_2_f (5’ CGCGGATCCCGGCGTGGTCAACGTC 3’) and CZ_2_r (5’TGCTCTAGACCCTGTCCGTTCATCNNN 3’), respectively. The kanamycin resistance cassette (*Kanr*) was amplified by PCR using the primer pairs kanf (5’ CCGGATATCTACAAGGGGTGTTATGAGCC 3’) and kanr (5’ CGCGGATCCTTAGAAAAACTCATCGAGCAT 3’). The final PCR products (*CZ*_*1*_: 993 bp, *CZ*_*2*_: 992 bp, *Kanr*: 832 bp) were cloned into the pMD19-T vector, and used for sequencing to confirm the correct amplification. Fragments CZ1 and CZ2 were used as the left bridge and the right bridge, respectively. Three resulting DNA fragments were digested by the restriction enzymes and inserted into the *Eco*RV and *Xba*I sites of the pKC1139 vector to generate pKC1139-CZ1-Kanr-CZ2 (pKCΔotrC). The recombinant plasmid was demethylated and introduced into either M4018 or SR16. The screening of the *otrC* disruption mutants M4018/pKCΔotrC and SR16/pKCΔotrC followed standard procedures [30]. Confirmation of the *otrC* disruption was performed by the PCR amplification of kanr using genomic DNA of the mutants as a template.

### Effect of OtrC expression on drug susceptibility

*E.coli*/pETC02 was grown in LB liquid medium with 50 μg/ml kanamycin, IPTG (1 mM) and different concentrations of drugs at 37°C, 170 rpm for 12 h, and then resuspended in 1 volume of fresh LB. Then, 50 μl cell suspension was spread immediately on the LB plates with 50 μg/ml kanamycin and 1 mM IPTG and different concentration of drugs, the plates were cultured at 37°C for 12 h to determine the minimal inhibitory concentrations (MICs). Experiments were performed in triplicate using *E. coli*/pET28a as the negative control.

*S. rimosus* mutants M4018/pSEC and M4018/pKCΔ*otrC* were grown in TSB adding different concentrations of tested drugs with M4018/pSET152 at 28°C and 220 rpm; after 30 h growth, cells were collected by centrifugation and resuspended in 1 volume of TSB. Then, 50 μl of fresh mycelium suspension was spread immediately on the TSB plates with different concentration of tested drugs. The MICs were measured after incubation for 3 d at 28°C, and experiments were performed in triplicate.

### Effect of OtrC expression on OTC production in *S .rimosus*

The mutants of M4018 and SR16 were cultured on the MS plates at 28°C for 3–5 d along with the parental strains; spores were collected and inoculated into GYCS medium to a final concentration of 1 × 10^6^ spores per ml, after they were cultured at 28°C on a rotary shaker (260 rpm) for 72 h as the seed culture. For an assessment of OTC production, 1% seed culture was transferred into SC medium and cultured at 30°C on a rotary shaker (260 rpm) for 7 d. The OTC productivity of *S. rimosus* strains were measured by high performance liquid chromatography analysis (HPLC). Then, 1 ml culture samples were centrifuged at 12,000 rpm for 10 min after the pH was adjusted to pH 1.5-1.7 using 9 mol/L HCl. The supernatants were filtered through a 0.22-μm filter (Millipore, Bedford, MA) and 10 μl samples were injected for analysis. Agilent 1100 HPLC system equipped with a 5-μm C18 (Kromasil, Sweden) column (4.6 by 200 nm) was employed for the HPLC analysis. The mixture of 60% H_2_O, 10% methanol, 20% acetonitrile and 10% phosphoric acid (2 mM) was used for the mobile phase and applied with a constant flow rate of 0.8 ml min^-1^ over 10 min. The OTC detection wavelength was 350 nm [27].

### qRT-PCR analysis of transcription levels

The transcription level of the otrC gene was analyzed by qRT-PCR as described previously [31]. *S. rimosus* cells were collected at different developmental stages during fermentation. Fresh tissues were used for total RNA extraction immediately using the AxyPrepTM multisource total RNA miniprep kit (Axygen, USA). The genomic DNA was removed by DNase I digestion, and the purity of the RNA samples was measured as the ratio of RNA concentration (ng/μl) to protein concentration (ng/μl) by Qubit^TM^ RNA Assay Kits and Qubit^TM^ Protein Assay Kits (Invitrogen, CA, USA), using the Qubit® 2.0 fluorometer (Invitrogen), according to the instructions provided by the manufacturer. RNA samples (RNA:protein ratio, 1.8-2.0) were selected and examined by 3% agarose gel electrophoresis to check their integrity prior to qRT-PCR analysis [32,33]. First strand cDNA synthesis was performed with a reverse transcription kit (TaKaRa, Japan) according to the manufacturer’s instructions. Standard curve was consisting of a 10-fold serial dilution series of five points which was prepared from cDNA samples (35 ng/μl) and each dilution was tested under a range of temperatures around the calculated Tm of the primers used in the experiments; untranscribed RNA samples were used as negative controls. The Cq values were determined for the optimization of qRT-PCR conditions.

qRT-PCR was assessed using SYBR® GC Premix Ex Taq^TM^ kits (TaKaRa, Japan) and a CFX96TM 168 real-time PCR detection system (Bio-Rad, USA). The total 25 μl reaction volume contained 1 μl DNA, 0.2 *μ*M forward primer, 0.2 μM reverse primer, and 1× SYBR® Premix Ex Taq^TM^, and the reaction conditions were as follows: 95°C for 30 s, and 40 cycles of 95°C for 5 s, 55-61°C for 30 s and 72°C for 30 s. Analysis of the melting curve was performed over a range of 55°C to 95°C for 5 s at the end of the PCR cycles. Three housekeeping genes, 16S rRNA, *hrdB* and G6PDH (*zwf1*) were used as reference genes to normalize the data [32,34]. The optimized Tm and cDNA concentration of each gene were used for qRT-PCR analysis. The primers for qRT-PCR were as follows: QotrCf (GTCACACGAGCGCCCTGGT) and QotrCr (CGCCGCCGAAGACGTACAC) were used for *otrC* transcriptional level analysis; QhrdBf (CTCTGTCATGGCGCTCA) and QhrdBr (ACGTTCTTCCACTGAGTGG) for the reference gene *hrdB*; Q16Sf (AGACACGGCCCAGACTC) and Q16Sr (CTGCTGAAAGAGGTTTACAAC) for 16S rRNA; Qzwff (ACTGGGCCAGAACGCCCT) and Qzwfr (AGTCCATCGAGACGTCCCGTA) for *zwf1*.

## Competing interests

The authors declare that they have no competing interests.

## Authors’ contributions

LY designed the approach, constructed the recombinant plasmids and mutants, and performed the ATPase activity and EB efflux assay, carried out bioinformatics research for conserved motifs analysis, prepared the draft the manuscript. XYY provided technical assistance with PCR amplification, plasmid identification and determine the minimal inhibitory concentrations of drugs. LW assisted with fermentation and OTC production measurement. MJG supervised LY, and participated in approach design, manuscript preparation. JC provided assistance on background studies, participated in manuscript preparation and editing. YPZ helped MIC determination and gave many useful suggestions. SLZ supervised LY for manuscript preparing and submitted it. All authors read and approved the final manuscript.

## Supplementary Material

Additional file 1 **Figure S1.** Construction of the recombinant plasmid pET28a-otrC (pETC02) for heterologous expression of OtrC in E. coli. **Figure S2.** ATPase assay of OtrC-overexpressing and OtrC-nonexpressing cells with different reaction time. **Figure S3**. Construction of the recombinant plasmid pSET152-Erme*p-*otrC* (pSEC) and identification of its integration in *S. rimosus.* Figure S4. Construction and identification of the recombinant plasmid pKC1139-*CZ*_*1*_-Kanr-*CZ*_*2*_ (pKCΔ*otrC*) for *otrC* disruption in *S. rimosus.* Click here for file

## References

[B1] Gustot A Smriti Ruysschaert JM McHaourab H Govaerts C Lipid composition regulates the orientation of transmembrane helices in HorA, an ABC multidrug transporter J Biol Chem 2010 285 19 14144 14151 10.1074/jbc.M109.079673 20223819 PMC2863179

[B2] Rodriguez-Garcia A Santamarta I Perez-Redondo R Martin JF Liras P Characterization of a two-gene operon *epeRA* involved in multidrug resistance in *Streptomyces clavuligerus* Res Microbiol 2006 157 6 559 568 10.1016/j.resmic.2005.12.008 16797928

[B3] Oliveira AS Baptista AM Soares CM Conformational changes induced by ATP hydrolysis in an ABC transporter: a molecular dynamics study of the Sav 1866 exporter Proteins 2011 79 6 1977 1990 10.1002/prot.23023 21488101

[B4] Choudhuri BS Bhakta S Barik R Basu J Kundu M Chakrabarti P Overexpression and functional characterization of an ABC (ATP-binding cassette) transporter encoded by the genes *drrA* and *drrB* of *Mycobacterium tuberculosis* Biochem J 2002 367 279 285 10.1042/BJ20020615 12057006 PMC1222852

[B5] Ward A Reyes CL Yu J Roth CB Chang G Flexibility in the ABC transporter MsbA: alternating access with a twist Proc Natl Acad of Sci USA 2007 104 48 19005 19010 10.1073/pnas.0709388104 18024585 PMC2141898

[B6] Zou P McHaourab HS Alternating access of the putative substrate-binding chamber in the ABC transporter MsbA J Mol Biol 2009 393 3 574 585 10.1016/j.jmb.2009.08.051 19715704 PMC2760602

[B7] Chang G Roth CB Structure of MsbA from *E. coli*: a homolog of the multidrug resistance ATP-binding cassette (ABC) transporters Science 2001 293 5536 1793 1800 10.1126/science.293.5536.1793 11546864

[B8] Linton KJ Structure and function of ABC transporters Physiology (Bethesda) 2007 22 122 130 10.1152/physiol.00046.2006 17420303

[B9] Linton KJ Higgins CF Structure and function of ABC transporters: the ATP switch provides flexible control PflügersArchiv European Journal of Physiology 2007 453 5 555 567 10.1007/s00424-006-0126-x 16937116

[B10] Grote M Polyhach Y Jeschke G Steinhoff HJ Schneider E Bordignon E Transmembrane signaling in the maltose ABC transporter MalFGK2-E: periplasmic MalF-P2 loop communicates substrate availability to the ATP-bound MalK dimmer J Biol Chem 2009 284 26 17521 17526 10.1074/jbc.M109.006270 19395376 PMC2719391

[B11] Loo TW Bartlett MC Clarke DM The "LSGGQ" motif in each nucleotide-binding domain of human P-glycoprotein is adjacent to the opposing Walker A sequence J Biol Chem 2002 277 41303 41306 10.1074/jbc.C200484200 12226074

[B12] Qiu J Zhuo Y Zhu D Zhou X Zhang L Bai L Deng Z Overexpression of the ABC transporter AvtAB increases avermectin production in *Streptomyces avermitilis* Appl Microbiol Biotechnol 2011 92 2 337 345 10.1007/s00253-011-3439-4 21713508

[B13] Malla S Niraula NP Liou KK Sohng JK Self-resistance mechanism in *Streptomyces peucetius*: Overexpression of *drrA*, *drrB* and *drrC* for doxorubicin enhancement Microbiol Res 2010 165 4 259 267 10.1016/j.micres.2009.04.002 19651502

[B14] McMurry LM Levy SB Revised sequence of *OtrB* (tet347) tetracycline efflux protein from *Streptomyces rimosus* Antimicrob Agents Chemother 1998 42 3050 9867793 10.1128/aac.42.11.3050 PMC105996

[B15] Petkovic H Cullum J Hranueli D Hunter IS Perić CN Pigac J Thamchaipenet A Vujaklija D Long PF Genetics of *Streptomyces rimosus*, the oxytetracycline producer Microbiol Mol Biol Rev 2006 70 704 728 10.1128/MMBR.00004-06 16959966 PMC1594589

[B16] Guilfoile PG Hutchinson R A bacterial analog of the *mdr* gene of mammalian tumor cells is present in *Streptomyces peucetius*, the producer of doxorubicin and daunorubicin Proceedings of the National Academy of Sciences USA 1991 88 8553 8557 10.1073/pnas.88.19.8553 PMC52547 1924314

[B17] Fernandez-Moreno MA Carbo L Cuesta T Vallin C Malpartida F A silent ABC transporter isolated from *Streptomyces rochei* F20 induces multidrug resistance J Bacteriol 1998 180 16 4017 4023 9696745 10.1128/jb.180.16.4017-4023.1998 PMC107393

[B18] Zhang H Pradhan P Kaur P The extreme C terminus of the ABC protein DrrA contains unique motifs involved in function and assembly of the DrrAB complex J Biol Chem 2010 285 49 38324 38336 10.1074/jbc.M110.131540 20876527 PMC2992266

[B19] Kaur P Rao DK Gandlur SM Biochemical characterization of domains in the membrane subunit DrrB that interact with the ABC subunit DrrA: identification of a conserved motif Biochemistry 2005 44 2661 2670 10.1021/bi048959c 15709779

[B20] Malla S Niraula NP Liou K Sohng JK Improvement in doxorubicin productivity by overexpression of regulatory genes in *Streptomyces peucetius* Res Microbiol 2010 162 109 117 20045726 10.1016/j.resmic.2009.12.003

[B21] Smanski MJ Peterson RM Rajski SR Shen B Engineered *Streptomyces platensis* Strains that overproduce antibiotics platensimycin and platencin Antimicrob Agents Chemother 2009 53 4 1299 1304 10.1128/AAC.01358-08 19164156 PMC2663125

[B22] Xu D Kim TJ Park ZY Lee SK Yang SH Kwon HJ Suh JW A DNA-binding factor, ArfA, interacts with the *bldH* promoter and affects undecylprodigiosin production in *Streptomyces lividans* Biochem Biophys Res Commun 2009 379 2 319 323 10.1016/j.bbrc.2008.12.052 19103157

[B23] Yuan LZ Rouviere PE Larossa RA Suh W Chromosomal promoter replacement of the isoprenoid pathway for enhancing carotenoid production in E. coli Metab Eng 2006 8 1 79 90 10.1016/j.ymben.2005.08.005 16257556

[B24] Steigedal M Valla S The Acinetobacter sp. chnB promoter together with its cognate positive regulator ChnR is an attractive new candidate for metabolic engineering applications in bacteria Metab Eng 2008 10 12 121 129 17950643 10.1016/j.ymben.2007.08.002

[B25] MacNeil DJ Gewain KM Ruby CL Dezeny G Gibbons PH MacNeil T Analysis of *Streptomyces avermitilis* genes required for avermectin biosynthesis utilizing a novel integration vector Gene 1992 111 61 68 10.1016/0378-1119(92)90603-M 1547955

[B26] Kieser T Bibb MJ Buttner MJ Chater KF Hopwood DA Practical Streptomyces genetics 2000 United Kingdom: John Innes Foundation, Norwich

[B27] Wang JY Yang SS Morphogenesis, biomass and oxytetracycline production of *Streptomyces rimosus* in submerged cultivation. annual scientific report of national laboratories of foods and drugs Depart of Health, Executive Yuan, Taiwan R.O.C 1996 14 68 78

[B28] Baykov AA Evtushenko OA Avaeva SM A malachite green procedure for orthophosphate determination and its use in alkaline phosphatase-based enzyme immunoassay Anal Biochem 1988 171 2 266 270 10.1016/0003-2697(88)90484-8 3044186

[B29] Sakamoto K Margolles A van Veen HW Konings WN Hop resistance in the beer spoilage bacterium *Lactobacillus brevis* is mediated by the ATP-binding cassette multidrug transporter HorA J Bacteriol 2001 183 18 5371 5375 10.1128/JB.183.18.5371-5375.2001 11514522 PMC95421

[B30] Bierman M Logan R O'Brien K Seno ET Rao RN Schoner BE Plasmid cloning vectors for the conjugal transfer of DNA from Escherichia coli to Streptomyces spp Gene 1992 116 1 43 49 10.1016/0378-1119(92)90627-2 1628843

[B31] Yu L Cao N Wang L Xiao C Guo M Chu J Zhuang Y Zhang S Oxytetracycline biosynthesis improvement in *Streptomyces rimosus* following duplication of minimal PKS genes Enzyme Microb Technol 2012 50 6–7 318 324 22500899 10.1016/j.enzmictec.2012.03.001

[B32] Taylor S Wakem M Dijkman G Alsarraj M Nguyen M **A practical approach to RT-qPCR**-**publishing data that conform to the MIQE guidelines** Methods 2010 50 S1 S5 10.1016/j.ymeth.2010.01.005 20215014

[B33] Bustin SA Benes V Garson JA Hellemans J Huggett J Kubista M Mueller R Nolan T Pfaffl MW Shipley GL Vandesompele J Wittwer CT The MIQE guidelines: minimum information for publication of quantitative real-time PCR experiments Clin Chem 2009 55 4 611 622 10.1373/clinchem.2008.112797 19246619

[B34] Li R Liu G Xie ZJ He XH Chen WQ Deng ZX Tan HR PolY, a transcriptional regulator with ATPase activity, directly activates transcription of polR in polyoxin biosynthesis in *Streptomyces cacaoi* Mol Microbiol 2010 75 2 349 364 10.1111/j.1365-2958.2009.06968.x 19919670

