# Supplementary material for: Molecular cloning and functional characterization of an ATP-binding cassette transporter OtrC from Streptomyces rimosus
Source: BMC Biotechnol. 2012 Aug 20;12:52. doi: 10.1186/1472-6750-12-52 (PMC3533511; doi:10.1186/1472-6750-12-52)
Supplement: Additional file 1 — Figure S1. Construction of the recombinant plasmid pET28a-otrC (pETC02) for heterologous expression of OtrC in E. coli. Figure S2. ATPase assay of OtrC-overexpressing and OtrC-nonexpressing cells with different reaction time. Figure S3. Construction of the recombinant plasmid pSET152-Erme*p-otrC (pSEC) and identification of its integration in S. rimosus. Figure S4. Construction and identification of the recombinant plasmid pKC1139-CZ1-Kanr-CZ2 (pKCΔotrC) for otrC disruption in S. rimosus. [file 1472-6750-12-52-S1.doc]

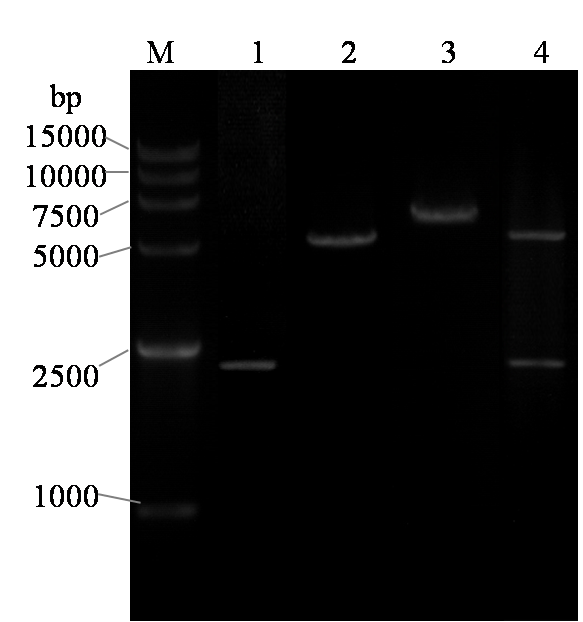


**Figure S1 Construction of the recombinant plasmid pET28a-*otrC* (pETC02) for heterologous expression of OtrC in *E. coli*.**

A: Agarose gel electrophoresis, DL15000 was used as DNA marker (M), recombinant plasmid pETC02 was identified by 0.8% agarose gel electrophoresis（lane 1）along with the empty vector pET28a (lane 2) as control. The o*trC* fragment (1913bp, lane 3) was amplified by PCR.

B: Identification of the recombinant plasmid pETC02, recombinant plasmid pETC02 was digested by *Nde* I and *Not* I (lane 4); *Bam* HI (7264bp, lane 5), pET28a was digested by Bam HI (5369bp, lane 6) for control.

**Figure S2 ATPase assay of OtrC-overexpressing and OtrC-nonexpressing cells with different reaction time.** *OtrC* from *S. rimosus* was introduced into *E.coli* BL21(DE3) using plasmid pETC02, and the *E.coli* BL21(DE3) carrying empty pET28a was used as control. The cells were induced by 1mM IPTG at 30ºC for10h, then collected by centrifugation, cell wall was digested by lysozyme and the membrane vesicles were harvested by centrifugation for ATPase activity determination. Vertical error bars correspond to the standard error of the mean of three replicated samples.

A


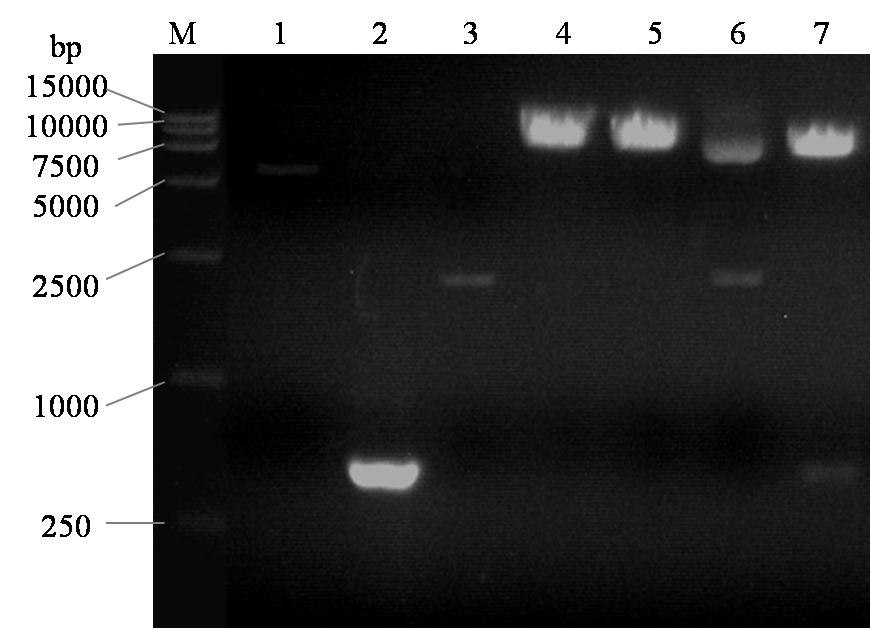


B


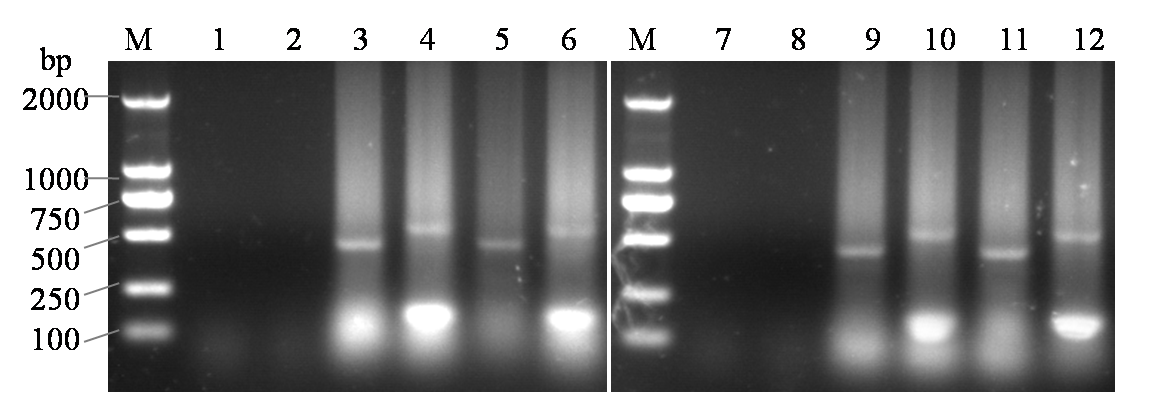


**Figure S3 Construction of the recombinant plasmid pSET152-*Erme*p*-*otrC* (pSEC) and identification of its integration in *S. rimosus***

A: Identification of the recombinant plasmid pSEC, DL15000 was used as DNA marker (M), recombinant plasmid pSEC was identified by 0.8% agarose gel electrophoresis（lane 1）；pSET152 was digested by *Eco*R I (5.7Kb, lane 2) for control, *Erme*p* fragment (484bp, lane3) and *otrC* fragment (1.8Kb, lane 4) were amplified by PCR, the recombinant plasmid pSEC was digested by *Eco*R I (lane 5) ; *Xba* I (lane5); *Bam*H I and *Xba* I (lane 6); *Eco*R I and *Bam*H I (lane 8), respectively.

B: Identification of the integration pattern. DL2000 was used as DNA marker (M), the *attL* (401bp ) and *attR* (502bp) fragments of M4018 (lane1 and lane 2), M4018/pSET152 (lane 3 and lane 4), M4018/pSEC (lane 5 and lane 6), SR16 (lane 7 and lane 8), SR16/ pSET152(lane 9 and lane 10) and SR16/pSEC (lane 11 and lane 12) were amplified by PCR, using the genomic DNA as templates.

A


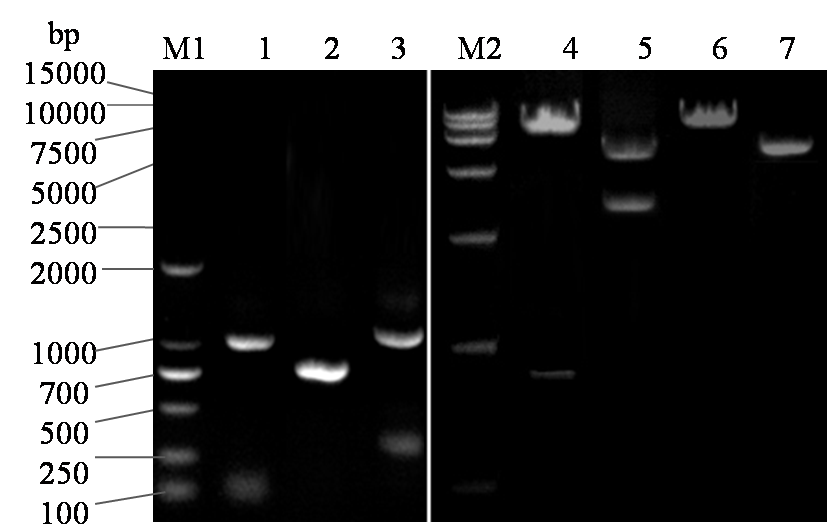


B


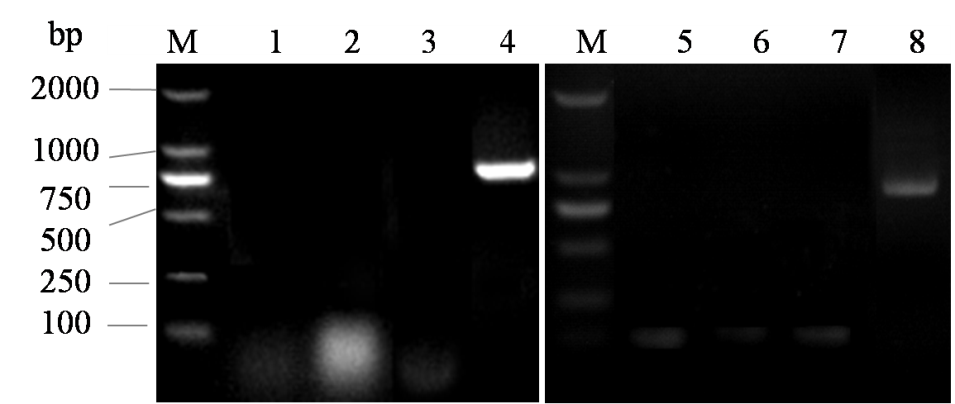


**Figure S4 Construction and identification of the recombinant plasmid pKC1139-*CZ_1_*-Kanr-*CZ_2_* (pKC△*otrC*) for *otrC* disruption in *S. rimosus***

A: construction and identification of recombinant plasmid pKC△*otrC*. DL2000 was used for DNA Marker (M), *CZ_1_* (lane 1), *Kanr* (lane 2) and *CZ_2_* (lane 3) fragments were amplified by PCR and used for construction of the recombinant plasmid pKC△*otrC*; and for identification, the recombinant plasmid pKC△*otrC* was digested by *Eco*R V and *Bam*H I (lane 4); *Eco*R I and *Xba* I (lane 5); *Eco*R I (lane 6); respectively, plasmid pKC1139 was digested by *Eco*R I(6.5Kb, lane 4) and used as control.

B: Identification of the double exchange pattern. DL2000 was used for DNA Marker (M), the *Aprr* and *Kanr* fragments of M4018 (lane 1 and 2), M4018/ pKC△*otrC* (lane 3 and 4), SR16 (lane 5 and 6) and SR/ pKC△*otrC* (lane 7 and 8) were amplified by PCR, using the genomic DNA as templates.
